# Scabies Treatment Failure: Beyond Drug Resistance and Toward a Multidimensional Understanding

**DOI:** 10.1155/jotm/1377975

**Published:** 2026-09-15

**Authors:** Jacob Al-Dabbagh

**Affiliations:** ^1^ Faculty of Medicine, Latakia University, Latakia, Syria; ^2^ Cancer Research Center, Latakia University, Latakia, Syria

## Abstract

Scabies, caused by *Sarcoptes scabiei var. hominis*, remains a major global health problem affecting both resource‐poor and high‐income regions. Despite the availability of effective treatments, treatment failure continues to pose a challenge to disease control and has become an increasingly recognized clinical and public‐health concern. However, treatment failure is often attributed solely to drug resistance, overlooking the complex interplay of biological, clinical, behavioral, and environmental factors. This narrative review summarizes the current state of knowledge regarding the multifactorial causes of scabies treatment failure. Key areas include true parasitological failure, pharmacological limitations, and emerging resistance; pseudoresistance resulting from incorrect treatment administration; reinfestation from untreated contacts; environmental transmission; diagnostic uncertainties; and host‐related inflammatory reactions such as postscabies pruritus. The review also proposes a multidimensional conceptual framework to distinguish true treatment failure from apparent clinical failure and to explain how interacting factors contribute to unsuccessful treatment outcomes. Identifying these determinants is crucial for optimizing treatment strategies, improving treatment success, and guiding future research aimed at combating scabies.

## 1. Introduction

Scabies continues to pose a significant global health burden; more than 200 million people are affected at any given time, with an estimated annual incidence of over 400 million cases [1]. Although the disease has traditionally been associated with resource‐poor regions, its classification as a neglected tropical disease in 2017 reflects its growing significance in both resource‐poor and high‐income regions, where an increase in incidence has been documented over the past decade [1–3]. Scabies, caused by *Sarcoptes scabiei var. hominis*, is frequently complicated by secondary bacterial infections, which can lead to serious consequences such as cellulitis, sepsis, and poststreptococcal glomerulonephritis [1, 4, 5].

Despite the availability of effective scabicide treatments, treatment failure remains a major clinical challenge [6]. Recent evidence suggests that treatment failure is not uncommon, while reported failures with permethrin have increased over time [2, 6, 7]. However, these clinical failures should not be equated automatically with biological drug resistance, as treatment outcomes may also be influenced by application errors, untreated contacts, reinfestation, and diagnostic uncertainty [8–10]. There remains a significant knowledge gap regarding the distinction between true biological resistance and apparent treatment failure caused by treatment‐ and host‐related factors [6, 8]. While previous systematic reviews have provided valuable quantitative estimates of treatment efficacy, a clinically oriented framework integrating pharmacological, procedural, transmission‐related, diagnostic, and host factors remains needed [6, 11]. Therefore, this narrative review summarizes current knowledge on the multifactorial causes of scabies treatment failure, with the aim of clarifying why standard therapies may appear ineffective in practice and providing a practical approach to distinguishing between true parasitological failure and apparent clinical failure.

## 2. Materials and Methods

A literature search was conducted in PubMed, Scopus, Web of Science, Semantic Scholar, and Google Scholar to identify relevant studies published between January 2020 and July 2026.

The search strategy combined Medical Subject Headings (MeSH) terms and free‐text keywords related to scabies and treatment outcomes, including “scabies,” “Sarcoptes scabiei,” “treatment failure,” “treatment resistance,” “permethrin resistance,” “ivermectin resistance,” “pseudoresistance,” “treatment adherence,” “reinfestation,” “contact management,” “environmental transmission,” “post‐scabies pruritus,” “persistent pruritus,” and “treatment outcomes.” Boolean operators (AND, OR) were applied in accordance with database‐specific requirements.

Priority was given to systematic reviews, meta‐analyses, observational studies, clinical guidelines, and consensus statements. Additionally, relevant clinical trials and key pharmacological, pharmacokinetic, diagnostic, or experimental/animal studies were incorporated when necessary to contextualize specific mechanisms, drug delivery, or host‐immune responses underlying treatment outcomes. Studies without relevance to the clinical, pharmacological, diagnostic, epidemiological, or biological aspects of human scabies management were excluded. Only English‐language publications were considered. Titles and abstracts were screened by the author, followed by full‐text assessment according to predefined eligibility criteria. The methodological limitations of included studies were considered qualitatively when interpreting the available evidence.

## 3. Distinguishing Between True Parasitological Failure and Apparent Clinical Failure

A major challenge in the current treatment of scabies is distinguishing between true parasitological failure and apparent clinical failure, as persistent symptoms are often mistakenly interpreted as a continuing infestation, leading to unnecessary repeat treatments and suffering for the patient [12–15]. For the purposes of this review, true parasitological failure refers to persistence of viable mites despite adequate treatment, whereas apparent clinical failure refers to persistent or recurrent symptoms that may occur with or without demonstrable active infestation, including those related to postscabies inflammation, inadequate treatment implementation, or reinfestation. This distinction provides the clinical basis for interpreting treatment outcomes throughout the review.

### 3.1. True Parasitological Treatment Failure

True parasitological treatment failure refers to persistence of viable mites after correctly administered and adequate therapy, with continued infestation demonstrated by appropriate clinical or parasitological assessment [8, 9]. Although reported treatment‐failure rates provide an important indication of reduced clinical effectiveness, they do not by themselves establish the presence of biological resistance [6, 8]. The distinction between clinical nonresponse and confirmed resistance is examined in detail in Section 4.

### 3.2. Pseudoresistance as a Cause of Apparent Clinical Failure

Pseudoresistance is an important cause of apparent clinical failure and refers to reduced treatment effectiveness resulting from factors unrelated to decreased mite susceptibility [8–10]. These factors include incorrect application, incomplete adherence, inadequate management of close contacts, and other procedural barriers that may prevent adequate drug exposure [2, 11]. Topical treatment is particularly vulnerable to such factors because effective therapy requires extensive whole‐body application and coordinated management of relevant contacts [11, 14]. The practical and behavioral determinants of these barriers are examined in greater detail in Section 6.1. Furthermore, the lack of a universally accepted method for confirming mite eradication complicates clinical assessment after treatment [2, 16].

### 3.3. Clinical Assessment of Persistent Symptoms After Treatment

Persistent pruritus after treatment should not, by itself, be interpreted as evidence of ongoing infestation [13]. The absence of new lesions, improvement in nocturnal itching, and progressive healing of existing lesions, particularly in the absence of viable mites or eggs on appropriate examination, support clinical resolution [17–19]. To prevent unnecessary scabicidal applications, patients must be counseled that posttreatment pruritus is often stubborn and debilitating, commonly persisting for several months after successful eradication with a reported median of 52.5 days [20, 21]. Conversely, the development of new burrows or demonstration of viable parasites after treatment should prompt further assessment for persistent infestation or reinfestation [17, 22, 23]. The inflammatory mechanisms and clinical course of postscabies pruritus (PSP) are discussed in Section 7.

## 4. Pharmacological Limitations and Emerging Evidence of Reduced Susceptibility

The efficacy of standard treatments for scabies is increasingly being questioned due to reported clinical failures and potential biological changes in susceptibility; however, clinical nonresponse should be distinguished from confirmed biological resistance [2, 9]. Although permethrin and ivermectin remain extremely valuable treatments, their apparent failures may result from pharmacological limitations, inadequate treatment implementation, or reinfestation, and therefore require systematic evaluation before reduced drug susceptibility is attributed [6, 9].

### 4.1. Hierarchy of Evidence in the Interpretation of Resistance to Scabicide Treatments

Evidence of reduced sensitivity to scabicides should be interpreted with caution, as clinical treatment failure is only an indirect indication of resistance and is often confounded by improper use, incomplete contact treatment, and reinfestation [8, 24]. Molecular resistance markers, including sodium channel mutations, remain insufficiently validated in *human Sarcoptes scabiei* populations and are based primarily on findings from canine models or related ectoparasites [2, 8]. Similarly, while laboratory susceptibility testing provides mechanistic insights, it has not consistently demonstrated reduced drug susceptibility in mites isolated from treatment‐resistant human cases [6, 8, 22]. Thus, resistance should be evaluated through a hierarchy of evidence: Clinical nonresponse should first be confirmed, alternative explanations should be excluded, and laboratory or molecular evidence should then be sought where available. Clinical nonresponse alone should not be regarded as confirmation of biological resistance [2, 8].

### 4.2. Permethrin Failure

Permethrin 5% cream remains a cornerstone therapy due to its proven efficacy and favorable safety profile [9, 23]. However, recent meta‐regression analyses suggest a progressive increase in reported clinical treatment failure rates, estimated at 0.58% annually between 1983 and 2021 [6]. Some European clinical centers have reported cure rates of only 27%–31%, although these findings are context‐specific and cannot be generalized globally [18, 22, 25].

The mechanisms underlying this reduced efficacy remain unclear. Proposed biological mechanisms include “knockdown resistance” mutations that affect voltage‐gated sodium channels, as well as enhanced detoxification pathways involving glutathione S‐transferase, esterases, and ABC transporters; however, most of the evidence comes from experiments or nonhuman models [2, 19, 26]. Furthermore, the “steal effect” hypothesis suggests that the binding of permethrin to keratin in hyperkeratotic skin could reduce the availability of the active ingredient and possibly contribute to subtherapeutic exposure [8].

The contribution of application‐related factors should be considered when interpreting these clinical failures, particularly because inadequate coverage and insufficient exposure time can reduce treatment effectiveness [11, 24].

### 4.3. Pharmacokinetics and Dosing Regimens of Oral Ivermectin

Following the WHO’s 2017 designation of scabies as a neglected tropical disease, oral ivermectin has been increasingly integrated into global disease control and mass drug administration programs [1, 9]. The standard dosing recommendation for oral ivermectin is 200 µg/kg administered in two doses, typically 7–14 days apart [9, 27]. Because ivermectin binds specifically to glutamatergic chloride (GluCl) channels in adult mites but lacks ovicidal activity, a single oral dose is pharmacologically limited, making a repeated dose on Day 14 necessary to prevent treatment failure [17].

A key discrepancy exists in clinical guidelines regarding coadministration with food [23]. Traditional manufacturer labels and national guidelines in countries like Spain and Turkey recommend administering ivermectin strictly after fasting (on an empty stomach) to prevent unpredictable absorption profiles [22, 23]. Conversely, newer recommendations, such as the European and US CDC guidelines, instruct patients to take ivermectin with food [9, 19]. This divergence is highlighted by substantial pharmacokinetic evidence, as co‐administering ivermectin with a high‐fat meal significantly enhances its oral absorption, bioavailability, maximum plasma concentration (*Cmax*), and area under the curve (AUC) [23, 28].

However, the clinical significance of this meal‐induced pharmacokinetic surge remains unproven, and evidence linking it to improved clinical outcomes is limited [23]. Although higher systemic levels could theoretically improve drug delivery to the skin, Nemecek et al. demonstrated that taking ivermectin with a meal was associated with a more than four‐fold increase in treatment failure compared to fasting [24].

Furthermore, escalating systemic concentrations of ivermectin could raise the risk of neurotoxicity without providing a proven therapeutic benefit, as higher blood levels may increase toxicity risks without significantly improving clinical efficacy [2, 23]. Ultimately, documented treatment failures must be carefully contextualized, distinguishing ivermectin’s inherent pharmacological limitations from biological drug resistance [2, 9]. While laboratory reports have suggested potential resistance pathways, including target‐site GluCl channel polymorphisms, cytochrome P450‐ or GST‐mediated metabolic detoxification, and P‐glycoprotein‐ or MRP3‐mediated efflux, confirmed clinical resistance remains insufficiently established [2, 9, 19, 27]. In clinical practice, inadequate dosing schedules, incomplete treatment, and reinfestation should be excluded before reduced drug susceptibility is considered.

## 5. Alternative Scabicidal Therapies and Their Clinical Role

When standard treatments, such as permethrin and oral ivermectin, are ineffective or unsuitable due to contraindications, intolerance, or suspected reduced susceptibility, alternative treatments may offer important therapeutic options [2, 9]. However, their clinical role is influenced not only by efficacy but also by safety, availability, treatment complexity, and adherence requirements [1, 9].

### 5.1. Benzyl Benzoate

Benzyl benzoate (10%–25%) remains one of the most widely used alternatives to permethrin and is often considered in situations where a decline in permethrin’s efficacy is suspected [9, 23]. Data from systematic reviews suggest that the overall treatment outcomes of benzyl benzoate and oral ivermectin are comparable across different follow‐up periods, while its low cost and global availability support its continued use, particularly in resource‐limited settings [1, 9].

Its main limitation is tolerability, as a burning sensation and irritation may reduce adherence to the treatment regimen, particularly in children and patients with inflammatory skin conditions [1, 9]. Compared to benzyl benzoate, ivermectin has a significantly lower incidence of local skin irritation [1].

### 5.2. Sulfur‐Based Preparations

Sulfur preparations (6%–33%) remain valuable options for population groups for whom other treatment options may be limited, particularly infants and pregnant and breastfeeding women [9, 17, 23]. Their low cost, widespread availability, and long‐term clinical experience support their continued use during disease outbreaks and among vulnerable populations [9, 29].

However, unpleasant odor, cosmetic discomfort, skin dryness, and the need for repeated daily application may compromise treatment adherence compared with simpler treatment regimens [9, 17, 29]. Although supported by extensive clinical experience, contemporary comparative evidence remains more limited than for first‐line treatment options [9, 17].

### 5.3. Crotamiton and Malathion

Crotamiton 10% is generally used in patients who cannot tolerate the preferred treatment methods, and it may provide additional symptomatic relief from itching [2, 9]. However, its efficacy is generally lower than that of permethrin or ivermectin, and repeated use may reduce treatment adherence [9, 17, 23].

Malathion 0.5% is a specialized alternative for cases resistant to first‐line treatment, although evidence supporting its efficacy in treating pruritus remains limited, and concerns regarding availability and safety have limited its wider use [9, 17].

### 5.4. New and Specialized Medications

Newer medications are being studied to overcome the limitations of current treatment methods. Spinosad 0.9% has shown promising efficacy even after a single application; clinical trials have reported cure rates of 78.1%–85%, although cost and limited availability remain barriers to widespread adoption [9, 22].

Moxidectin is a potential future systemic treatment option, as it has a longer half‐life and greater tissue persistence compared to ivermectin; however, its safety and efficacy in the treatment of human scabies must be confirmed by larger clinical trials [2, 9, 17]. In contrast, 1% lindane is no longer recommended due to concerns regarding neurotoxicity [9, 19].

The comparative characteristics and clinical roles of available and new scabies treatment methods are summarized in Table 1 [1, 2, 9, 17, 19, 22–24, 29–33].

**TABLE 1:** Comparative pharmacological profiles, clinical efficacy, and therapeutic roles of established and emerging antiscabietic agents.

Treatment method (route)	Dosing regimen and administration	Clinical efficacy (cure rates)	Special populations (pregnancy/pediatrics)	Key advantages and major limitations
Permethrin 5% (Topical Cream/Lotion)	Apply head‐to‐toe nocturnal application; wash off after 8–12 h (4‐5 h in infants is recommended). Repeat after 7–14 days.	Historical: High (∼90%–95%+), but real‐world clinical failure rates are rising. Meta‐regression: Shows a significant annual increase in failure rate (+0.58% per year).	Pregnancy: Category B (safe, LoE III, Grade B). Lactation: Considered safe. Pediatrics: Approved for infants ≥ 2 months of age.	Advantages: Highly effective, low systemic toxicity. Limitations: Transient local irritation; high rates of pseudoresistance due to application errors.

Oral Ivermectin (Systemic Tablets)	200 µg/kg in two separate oral doses, given 7–14 days apart. Take with food (per European/CDC guidelines) to increase absorption or after fasting for 2 h (per Turkish guidelines).	Two‐dose regimen: Failure rate is very low (4.2% vs. 13.1% for a single dose). Overall: Cure rates are highly comparable to permethrin.	Pregnancy: Contraindicated. Lactation: Generally avoided; safety not fully established. Pediatrics: Approved only for children ≥ 15 kg (or ≥ 5 years).	Advantages: Ease of administration (highly improves compliance); ideal for outbreaks and MDA. Limitations: Not ovicidal (demands a second dose); neurotoxicity risk at high levels.

Benzyl Benzoate 10%–25% Topical Lotion)	Apply once daily at night for two successive days; wash off after 24 h. Reapply after 7 days. Dilute to 10% for children; use 25% for adults.	Comparative: Comparable cure rates to oral ivermectin at 2–4 weeks (82.4% for BB vs. 78.7% for IVM, RR 0.99).	Pregnancy: Safe under European guidelines (LoE III, Grade B), but avoided under Turkish guidelines. Lactation: Avoided/not recommended. Pediatrics: 10% concentration for children aged 1–5 years.	Advantages: Extremely low cost, widely available, highly cost‐effective. Limitations: Severe skin burning, stinging, and irritant contact dermatitis, leading to high nonadherence.

Sulfur Preparations 6%–33% (Topical Ointment)	Apply once daily for three consecutive days; wash off 24 h after the last application.	Efficacy: Decades of empirical use, but high‐quality contemporary comparative trial evidence remains limited compared to first‐line therapies.	Pregnancy: Category C (probably safe, used at 6% concentration). Lactation: Probably safe at 6%. Pediatrics: Safe for infants < 2 months and neonates.	Advantages: Cheap, widely accessible, safe for neonates and pregnant women. Limitations: Unpleasant odor, cosmetic messiness, clothing staining, and risk of xerotic eczema (up to 33% of patients).

Crotamiton 10% (Topical Cream/Lotion)	Apply once daily for 3–5 consecutive days; wash off 24 h after the last application.	Efficacy: Significantly lower therapeutic efficacy compared to standard first‐line therapies.	Pregnancy: Category C (use with caution). Lactation: Safety unknown. Pediatrics: Can be used in infants with caution.	Advantages: Mild antipruritic effect provides symptomatic relief from itching. Limitations: Low cure rates and highly complex regimen (3–5 days of daily application).

Malathion 0.5% (Topical Aqueous Lotion)	Apply once weekly for 2 weeks; leave in contact with skin for 24 h before washing off.	Efficacy: Moderate efficacy; supported by very limited contemporary comparative evidence.	Pregnancy: Not recommended. Lactation: Safety and excretion into breast milk unknown. Pediatrics: Safety not established.	Advantages: Specialized alternative for cases refractory to standard first‐line treatments. Limitations: Extremely limited global availability; withdrawn from several markets due to safety concerns.

Spinosad 0.9% (Topical Suspension)	Single topical application; apply head‐to‐toe, leave on skin for 6 h (or overnight), then wash off.	Efficacy: Excellent efficacy from a single dose; RCTs report a 78.1% complete clinical cure rate and 85% microscopic/dermatoscopic cure rate at Day 28.	Pregnancy: Safety profile under‐studied. Lactation: Safety unknown. Pediatrics: Approved for pediatric patients ≥ 4 years old.	Advantages: High cure rate from a single, short‐contact application (6 h), greatly enhancing compliance. Limitations: High cost and limited global geographic availability.

Oral Moxidectin (Emerging/Experimental)	Single oral dose (typically 0.3 mg/kg under experimental/onchocerciasis protocols). Human trials (NCT03905265) are ongoing.	Efficacy: Superior single‐dose efficacy compared to ivermectin in porcine models, achieving complete parasite clearance. Human trials are underway.	Pregnancy: Safety not established. Lactation: Safety unknown. Pediatrics: Under clinical investigation.	Advantages: Superb pharmacokinetics: very long half‐life of ∼23 days (covers entire mite life cycle in a single dose) and superior skin penetration. Limitations: Human safety and efficacy trials for scabies are still ongoing.

## 6. Behavioral and Practical Aspects: Factors Influencing Treatment Success

The success of scabies treatment depends not only on the effectiveness of medications but also on the precise implementation of treatment strategies [2, 11]. Apparent treatment failure often results from the interaction of patient, social, and environmental factors that hinder the effective eradication of the itch mite and promote reinfestation [6, 22].

### 6.1. Patient‐Related Factors: Barriers to Treatment Implementation and Adherence Issues

Barriers to medication use are a major determinant of treatment outcomes, particularly for topical treatments that require extensive and proper coverage of the entire body [8, 24].

Objective assessments using fluorescent‐labeled creams have demonstrated significant difficulties in achieving complete drug coverage; in one prospective study, no participant was able to achieve adequate whole‐body coverage, with a median of six untreated body parts [24].

In a separate individual multicenter cross‐sectional study, inadequate application, failure to reapply medication after handwashing, and failure to read the accompanying treatment instructions were significantly associated with treatment failure [11].

Multivariate regression analysis in that study showed that failure to apply topical medication to the entire body, including the interdigital and genital areas, was associated with treatment failure (OR 14.33, 95% CI: 2.875–71.426) [11]. Failure to reapply topical medication to the hands after washing was also associated with treatment failure (OR 3.47, 95% CI: 1.516–7.978), while failure to read the provided written step‐by‐step information sheet was associated with treatment failure (OR 13.67, 95% CI: 1.321–80.555) [11].

Additional barriers include uncertainty regarding the required amount of topical medication, with approximately 38% of patients reporting that a standard 30‐g tube was not sufficient for complete application, which may result in the omission of asymptomatic areas [24]. Local side effects, including irritant dermatitis, may further reduce treatment adherence and contribute to premature discontinuation of therapy [8].

### 6.2. Social Factors: Contact Management and the Dynamics of Reinfestation

Reinfestation represents an important cause of apparent treatment failure and may occur when close contacts remain untreated or when transmission persists within household and social networks [2, 14, 32]. Because infected individuals may remain asymptomatic during the incubation period, treatment of the index patient alone may not interrupt transmission [14, 16]. Synchronous management of relevant close contacts is therefore an essential component of scabies control [2, 14, 32].

Clinical evidence indicates that household‐level exposure, a family history of scabies or skin diseases, and the sharing of beds or clothing items are associated with a high frequency of scabies reinfestation, supporting the role of the domestic environment as an important transmission setting [31]. Overcrowding, low educational levels, and personal hygiene factors, such as infrequent bathing habits, have also been associated with increased disease severity and reinfestation frequency [31]. Stigma, inadequate contact tracing, and incomplete treatment of exposed contacts may further sustain transmission, particularly in densely populated households [12, 14].

Financial barriers may also impede timely treatment and adequate coverage of close contacts, particularly among vulnerable and low‐income populations [34]. In Italy, regional public‐health interventions in the Autonomous Province of Trento and Emilia‐Romagna have provided free scabies medications to cases and close contacts [34]. SCAB‐net was subsequently established as a multicenter registry protocol to collect standardized clinical data, evaluate treatment efficacy, and identify risk factors for treatment failure across public dermatology services; definitive treatment‐effectiveness results are not yet reported in this source [34].

Long‐term surveillance in the Bologna metropolitan area from 2013 to 2024 demonstrated a significant upward trend in scabies cases, showing a major re‐emergence of the disease, particularly in the latest years and during colder months [35]. Beyond the household, population mobility, including traveler and migrant movement, may contribute to repeated exposure and seasonal variation in transmission [35]. Overtourism and the growth of short‐term accommodations have also been proposed as potential contributors in some urban settings by increasing population mixing and opportunities for close contact in overcrowded or suboptimal living environments [35].

### 6.3. Environmental Factors: Risk of Reinfestation and Appropriate Disinfestation

The epidemiological significance of environmental reservoirs and fomites depends heavily on the clinical variant and the severity of the infestation [2, 17]. For common scabies, transmission occurs predominantly through prolonged, direct skin‐to‐skin contact, whereas indirect transmission through contaminated bedding, clothing, or other fomites is considered uncommon and of minor importance in classic disease [14, 16, 22].

In these typical cases of common scabies, the host mite burden is small, typically involving only 10–15 mites, making fomite‐mediated transmission very rare; indeed, studies have demonstrated that less than 1% of individuals exposed to contaminated bedding used by classic scabies patients contract the infestation [2, 23]. Under typical indoor conditions, scabies mites generally survive only 24–36 h away from a host, and their infectivity and ability to invade a new host decline rapidly over time [2, 9, 20]. Although longer survival times have been observed under certain cool or humid laboratory conditions, these experimental results should not be directly extrapolated to typical residential settings [2, 3, 20].

In contrast, crusted scabies represents a distinct epidemiological situation; the substantially higher mite burden, which can reach thousands to millions of parasites on a single host, and the active shedding of mite‐laden scales and crusts significantly increase the potential for environmental contamination and indirect transmission [2, 9, 36]. Consequently, environmental‐control measures, such as proper laundering and disinfestation of textiles, are particularly important in managing cases of crusted scabies and in preventing widespread transmission within institutional or outbreak settings [2, 9, 36].

Prospective observational data show that implementing environmental measures, such as daily changes of bed linens or sealing clothing in plastic bags for 4 days, is associated with improved treatment outcomes [18]. Environmental decontamination should therefore be viewed as a complementary control measure whose importance varies according to the clinical form and epidemiological setting. It should be prioritized particularly in crusted scabies and institutional or outbreak settings rather than applied as an extensive decontamination strategy in every case of common scabies [2, 14, 22, 36].

## 7. Host‐Related Factors and the Clinical Interpretation of PSP

Host‐related responses represent a distinct contributor to apparent treatment failure because inflammatory symptoms may persist after successful mite eradication [9, 13]. Rather than indicating ongoing parasitic activity, these symptoms may reflect the biological response of the host to residual mite‐derived antigens and subsequent cutaneous inflammation [2, 13]. PSP is therefore considered in this review as a major host‐related mechanism of apparent clinical nonresponse.

### 7.1. PSP: Definition and Duration

PSP refers to persistent itching following the biological resolution of a scabies infestation [13]. Although it has traditionally been considered a short‐term, self‐limiting phase lasting two to 4 weeks, recent clinical evidence indicates that symptoms may persist for significantly longer [13, 21]. Reported cohorts have documented mean or median symptom durations of approximately 52.5–56 days, with some patients experiencing symptoms for several months or, in individual cases, up to one year despite confirmed eradication of the infestation [13, 21].

The pathophysiology of PSP is incompletely understood and is likely multifactorial. Persistent inflammatory and immunological responses may be triggered by residual mite antigens, carcasses, eggs, and fecal material (scybala) retained within the stratum corneum after mites have been eradicated [2, 13, 15]. In active scabies, Th2‐skewed immune responses involving IL‐4 and IL‐13 have been associated with IgE production and eosinophilic infiltration, while IL‐31 may contribute to persistent itch [9, 13, 37]. Histopathological studies of postscabietic nodules have demonstrated lymphocytic and eosinophilic infiltrates, supporting a persistent hypersensitivity component [13, 29].

Neuroimmune mechanisms may also contribute to prolonged symptoms. Experimental evidence implicates pathways involving substance P, NK‐1R, PAR‐2, and MRGPRX2, together with alterations in peripheral sensory nerve function [37]. However, much of this mechanistic evidence derives from active scabies or experimental models, and its specific contribution to human PSP remains uncertain [13, 37].

### 7.2. Clinical Impact of PSP and Risk of Overtreatment

The clinical significance of PSP lies in its potential to distort the interpretation of treatment response [2, 13]. Persistent inflammation may lead patients and clinicians to assume that viable mites remain present, particularly when pruritic nodules persist after treatment [2, 13]. This may result in repeated courses of scabicidal therapy despite biological eradication, potentially aggravating skin irritation and perpetuating the perception of treatment failure [2, 13]. Persistent reddish‐brown nodules have been observed in 55.8% of individuals with long‐term symptoms and may be confused with active nodular scabies [13, 17, 23]. In addition, prolonged symptoms may contribute to psychological distress, including fear of persistent infestation and anxiety related to itching [2, 13]. Recognition of PSP as a posttreatment inflammatory phase is therefore critical for preventing unnecessary retreatment and directing clinical management toward appropriate symptomatic therapy and patient reassurance [13].

### 7.3. Host Factors Affecting Symptom Severity

Individual host characteristics may influence the severity and duration of posttreatment symptoms. A history of atopic predisposition and house dust mite allergy has been considered a potential risk factor for the development of more severe PSP, which may be associated with increased itch sensitivity and altered Th2‐mediated immune responses [13]. Advanced age may also be associated with prolonged symptoms, potentially due to immunosenescence and a reduced capacity for epidermal barrier repair, although further studies are needed to confirm this association [21]. These potential host‐related differences further support individualized counseling and follow‐up after treatment, particularly when symptoms persist in the absence of objective evidence of active infestation [2, 13, 23].

## 8. A Multidimensional Framework for Interpreting Persistent or Recurrent Symptoms After Scabies Treatment

Treatment failure in scabies should be interpreted as an outcome rather than as a single mechanism. Based on the evidence discussed in the preceding sections, this review proposes a multidimensional framework comprising five interacting domains: diagnostic uncertainty, pharmacological limitations and possible changes in susceptibility, procedural barriers, reinfestation and transmission dynamics, and host inflammatory responses [6, 22]. The framework is intended to explain why persistent or recurrent symptoms may represent true parasitological failure, apparent clinical nonresponse, or reinfestation rather than drug resistance alone.

Rather than functioning as independent pathways, these domains may overlap and reinforce one another. For example, diagnostic uncertainty may lead to inappropriate treatment, inadequate implementation may result in persistent infestation, untreated contacts may cause reinfestation, and postscabies inflammation may obscure the distinction between eradication and ongoing disease. The framework therefore emphasizes sequential clinical assessment before attributing nonresponse to reduced drug susceptibility.

**FIGURE 1:**
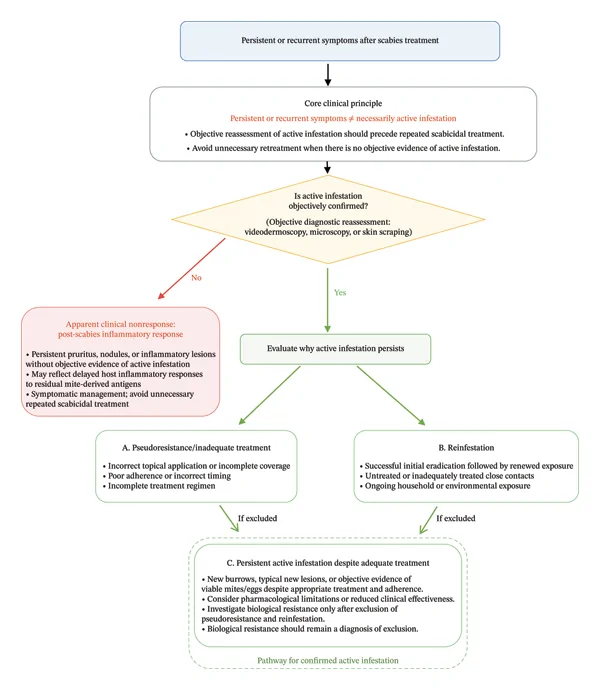
Conceptual framework of the multifactorial determinants of scabies treatment failure, including diagnostic, pharmacological, procedural, transmission, and host inflammatory factors.

To integrate these determining factors, the proposed framework illustrates the principal pathways through which treatment outcomes may diverge after therapy (Figure 1) [2, 6, 8, 9, 11, 12, 14–16, 20, 22, 24, 27, 32].

### 8.1. Diagnostic Uncertainty and Assessment of Parasite Eradication

Diagnostic uncertainty represents the first component of the framework because persistent or recurrent symptoms cannot be classified as treatment failure without establishing whether active infestation is still present [12]. As discussed in Section 3, the distinction between active infestation and PTI symptoms is essential for determining whether an apparent clinical failure represents true parasitological persistence. A misdiagnosis, such as eczema or atopic dermatitis, can delay the initiation of appropriate treatment and contribute to the development of atypical symptoms alongside an increased mite burden [20, 36]. Furthermore, the absence of a validated, noninvasive method to confirm post‐treatment clearance makes it difficult to distinguish persistent infestation from residual symptoms [2, 22]. Within the proposed framework, diagnostic uncertainty therefore represents the initial classification step rather than an independent mechanism of pharmacological failure.

### 8.2. Pharmacological Limitations and Emerging Changes in Susceptibility

Pharmacological factors contribute to treatment failure through the limitations of existing drugs and a potential decrease in mite susceptibility [6]. As detailed in Section 4, these possibilities should be interpreted only after clinical nonresponse has been confirmed and major alternative explanations, including inadequate treatment implementation and reinfestation, have been excluded [6, 8, 9]. Possible biological resistance therefore remains a diagnosis of exclusion, particularly because molecular and laboratory evidence in human scabies remains limited [2, 8].

### 8.3. Procedural Errors and Pseudoresistance

Pseudoresistance represents an important potentially reversible cause of apparent treatment failure. The application and adherence barriers described in Section 6.1 demonstrate that inadequate treatment implementation may reduce effective drug exposure despite the use of an appropriate scabicide [11, 24]. Within the framework, these factors should therefore be assessed before treatment is classified as pharmacological failure or reduced drug susceptibility.

### 8.4. Reinfestation and Social Transmission Dynamics

Reinfestation represents a distinct pathway to apparent treatment failure and should be differentiated from persistence of the original infestation [2, 14, 32]. As discussed in Section 6.2, untreated close contacts and ongoing household or social transmission may result in repeated exposure after apparently successful treatment of the index patient [14, 32]. Environmental transmission may provide an additional pathway in selected settings, particularly crusted scabies and institutional outbreaks, but is less important in common scabies [9, 14, 36]. Within the proposed framework, reinfestation should therefore be considered before attributing recurrent symptoms to inadequate drug efficacy or biological resistance.

### 8.5. Host Inflammatory Response and False Treatment Failure

The final component of this framework is the host’s immune response, which may mimic a persistent infestation despite successful eradication [2, 13, 22]. PSP and persistent nodular hypersensitivity should therefore be considered forms of apparent clinical nonresponse rather than parasitological failure once active infestation has been objectively excluded [13, 21]. Within the proposed multidimensional framework, this domain underscores the clinical necessity of interpreting persistent symptoms in the context of the patient’s overall clinical timeline and treatment history rather than relying on pruritus alone as a binary marker of therapeutic failure [2, 13].

## 9. Conclusion

Treatment failure in scabies is a multifactorial phenomenon resulting from the interaction of biological, procedural, diagnostic, transmission‐related, and host factors rather than drug resistance alone. Although rising treatment failure rates require ongoing monitoring, current evidence indicates that persistent or recurrent symptoms may arise from several distinct mechanisms, including inadequate treatment implementation, reinfestation, diagnostic uncertainty, and PTI responses, while confirmed biological resistance remains insufficiently established.

Successful treatment therefore requires an integrated approach that combines appropriate pharmacological therapy and dosing with effective implementation, concurrent management of relevant contacts, and accurate assessment of posttreatment symptoms. A multidimensional clinical approach may help reduce unnecessary retreatment, improve recognition of genuine treatment failure, and provide a more appropriate basis for investigating possible drug resistance. Further research should prioritize standardized definitions of treatment failure, objective methods for confirming mite eradication, prospective evaluation of treatment effectiveness, and validated approaches for detecting clinically relevant drug resistance.

## Funding

This research received no specific grant from any funding agency in the public, commercial, or not‐for‐profit sectors.

## Conflicts of Interest

The author declares no conflicts of interest.

## Data Availability

All data supporting this review are available in the included published studies. No new datasets were generated or analyzed.
